# A computational approach to identify cellular heterogeneity and tissue-specific gene regulatory networks

**DOI:** 10.1186/s12859-018-2190-6

**Published:** 2018-06-07

**Authors:** Ankit Jambusaria, Jeff Klomp, Zhigang Hong, Shahin Rafii, Yang Dai, Asrar B. Malik, Jalees Rehman

**Affiliations:** 1 Department of Pharmacology, The University of Illinois College of Medicine, 835 S. Wolcott Ave. Rm. E403, Chicago, IL 60612 USA; 2 Division of Regenerative Medicine, Department of Medicine, Ansary Stem Cell Institute, Weill Cornell Medicine, New York, NY USA; 3 Department of Bioengineering, The University of Illinois at Chicago, Chicago, IL USA; 4 Division of Cardiology, Department of Medicine, The University of Illinois College of Medicine, Chicago, IL USA

**Keywords:** Gene set enrichment, Systems biology, Tissue specificity, Gene expression, Transcriptional networks, Transcription factor binding motifs, Pathway analysis, Therapeutic targets, Endothelial cells, Endothelial heterogeneity, Neurons, Neuronal heterogeneity, Vascular biology

## Abstract

**Background:**

The heterogeneity of cells across tissue types represents a major challenge for studying biological mechanisms as well as for therapeutic targeting of distinct tissues. Computational prediction of tissue-specific gene regulatory networks may provide important insights into the mechanisms underlying the cellular heterogeneity of cells in distinct organs and tissues.

**Results:**

Using three pathway analysis techniques, gene set enrichment analysis (GSEA), parametric analysis of gene set enrichment (PGSEA), alongside our novel model (HeteroPath), which assesses heterogeneously upregulated and downregulated genes within the context of pathways, we generated distinct tissue-specific gene regulatory networks. We analyzed gene expression data derived from freshly isolated heart, brain, and lung endothelial cells and populations of neurons in the hippocampus, cingulate cortex, and amygdala. In both datasets, we found that HeteroPath segregated the distinct cellular populations by identifying regulatory pathways that were not identified by GSEA or PGSEA. Using simulated datasets, HeteroPath demonstrated robustness that was comparable to what was seen using existing gene set enrichment methods. Furthermore, we generated tissue-specific gene regulatory networks involved in vascular heterogeneity and neuronal heterogeneity by performing motif enrichment of the heterogeneous genes identified by HeteroPath and linking the enriched motifs to regulatory transcription factors in the ENCODE database.

**Conclusions:**

HeteroPath assesses contextual bidirectional gene expression within pathways and thus allows for transcriptomic assessment of cellular heterogeneity. Unraveling tissue-specific heterogeneity of gene expression can lead to a better understanding of the molecular underpinnings of tissue-specific phenotypes.

**Electronic supplementary material:**

The online version of this article (10.1186/s12859-018-2190-6) contains supplementary material, which is available to authorized users.

## Background

Computational analysis of microarray and RNA-Seq gene expression data of a given cell type obtained from distinct organs and tissues enables the unbiased identification of gene candidates. Endothelial cells, neurons, macrophages or fibroblasts that reside in different tissues or organs have distinct functions are thus likely to have unique gene expression signatures that reflect their tissue-specific adaptation and function. Several recent publications addressing cellular heterogeneity have gained widely applicable biological insight in many areas including disease subtypes [1], candidate biomarkers [2], and molecular mechanisms of disease [3]. After generating gene expression data for different tissues, the goal is to identify the molecular heterogeneity characterizing the tissues. The heterogeneous populations may represent previously unidentified molecular profiles responsible for tissue-specific function. Additionally, by studying patterns of gene expression in different tissues, insights into the regulatory landscape of each population can be obtained. To characterize heterogeneous or differentially regulated genes, differential expression analysis [4] and gene co-expression network analysis [5] are commonly used. A limitation of these methods however is that the single dimensional analysis of genes does not identify the causal molecular mechanisms that regulate them [6]. These methods rely on ranking individual genes by differential expression and subsequently inferring the underlying pathways or transcription factors that maintain the heterogeneous gene expression profiles. Importantly, deriving the initial rank-list of the most differentially expressed genes using these conventional computational approaches does not consider whether differential expression of tissue-specific genes is concentrated within functional groups. From a biological perspective, functionally related genes often have similar expression patterns which match cell-specific phenotypes [7]. In order to identify the molecular signature of distinct cell populations, new methods, in addition to existing methods such as GSEA [8] and PGSEA [9], need to be developed to interpret dynamic changes within a group of genes with common function. We therefore developed a novel unbiased computational approach (HeteroPath) in which cell type specific gene expression was analyzed from distinct tissues within the context of pathways. Instead of ranking pathways by cumulative gene expression of all individual genes, we ranked the pathways from most heterogeneous to least heterogeneous, and applied this approach in two cell types: endothelial cells and neurons that were freshly isolated from distinct tissues. This novel method of characterizing cells from distinct tissues based on the heterogeneity of the pathways allows for the precise identification of pathways which are uniquely upregulated or uniquely downregulated in a given tissue.

Endothelial cells lining the intimal of vessels in different organs have specialized functions and morphological features [10]. Heterogeneous endothelial cell (EC) populations also have distinct signature gene expression patterns, which allow for tissue-bed specific functional adaptation of the vasculature [11]. For example, microvascular endothelial cells in the brain form a continuous, highly impermeable blood-brain barrier (BBB) to maintain the selective metabolic balance required for brain function and restrict BBB permeability [12]. The lung endothelium, on the other hand, forms a semipermeable barrier which is apposed to the alveolar epithelium, thereby facilitating alveolar gas exchange which is essential for maintaining lung fluid balance [13]. The endothelial cells lining the heart vasculature also maintain a restrictive endothelial barrier while being exposed to intense shear forces generated by the contracting heart [11].

Similarly, neuronal specialization exists in different regions of the brain due to their unique morphology, connectivity, and electrophysiological properties [14]. It is thought that this heterogeneity arose during evolution for the “division of labor” and execution of specialized tasks in the mammalian nervous system [15]. Even though signature genes and proteins for distinct neuronal subpopulations have been established, a comprehensive transcriptomic analysis that would identify signature pathways for each subpopulation would likely help uncover novel subpopulation-specific functions and mechanisms.

In the case of endothelial cells and neurons, cellular heterogeneity is likely dictated by the niche and rooted in the tissue-specific regulation of gene expression pathways. Therefore, identifying tissue-specific gene expression signatures is necessary to define the phenotypic heterogeneity of the endothelium and of neurons.

## Methods

### Microarray data preprocessing

**Fig. 1:**
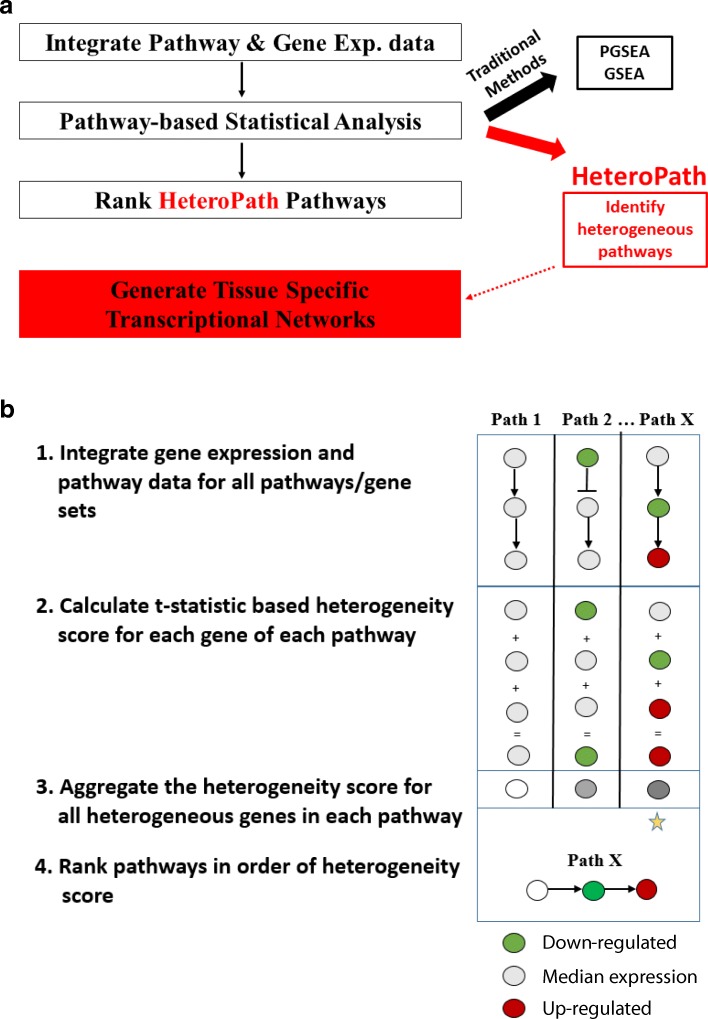
**a** Tissue-specific transcriptomic profiling**.** First, the gene expression data is preprocessed and normalized. Then, the gene expression data and gene set data are integrated together. Each KEGG pathway is statistically evaluated using the traditional algorithms GSEA, PGSEA, and the novel HeteroPath algorithm to identify tissue-specific pathways. Next, the tissue-specific gene regulatory networks are constructed by identification of heterogeneous genes and their regulatory transcription factors as determined by motif enrichment analysis using the ENCODE database. **b** The HeteroPath algorithm for identifying heterogeneous pathways and gene sets. HeteroPath aims to find the pathways/gene sets that are not only differentially expressed from the global median gene expression value but also appear to be responsible for the regulation of distinct cell types

The statistical modeling algorithm we developed (Fig. 1) was applied to microarray data sets downloaded from the Gene Expression Omnibus (GEO) at http://www.ncbi.nlm.nih.gov/geo/. The mouse endothelial cells (GSE47067) were freshly isolated from mouse organs, which were intravitally labeled, isolated via flow sorting and immediately processed for RNA extraction, amplification and hybridization [4]. The mouse forebrain neurons (GSE2882) were fluorescently labelled neurons isolated from five different regions of the forebrain. Datasets of three of the endothelial cell tissues (brain, lung, and heart) and three of the neuronal cell tissues (hippocampus, cingulate cortex, and amygdala) were used in this study.

After downloading the raw individual Affymetrix Mouse Gene 1.0 ST Array CEL files, data analysis was performed using software available from the Bioconductor Project (*version 3.2*) and the R Project for Statistical Computing v. 3.2.1 [16, 17]. We performed the background correction and the normalization of the raw expression data using the robust multi-chip average (RMA) from the *affy* package (1.24.2).

### HeteroPath algorithm

1. For each of the *N* genes in the gene expression matrix, calculate the t-statistic for each tissue by performing an individual-gene analysis:

$$ {t}_i=\frac{M_1(i)-{M}_2(i)}{s(i)} $$

Where *M*_1_(*i*) is the median expression level of gene *i* in an individual tissue, *M*_2_(*i*) is the median expression level of gene *i* across all tissues, and *s*(*i*) is a pooled standard deviation over the two groups (individual tissue vs median of all tissues).

2. Filter out genes which have less than a threshold for fold-change (The value 2 is set as default).
3. Compute the Heterogeneity score (HS) corresponding to pathway/set *S*:

$$ HS=\sum \limits_{i\in S}\left|{t}_i\right| $$

4. Permute the labels of the phenotype *P* in the data matrix and repeat (1) and (2). Repeat until all permutations are considered.
5. Compute empirical *p*-value for the association of *S* and *P* as the fraction of the HSs from the permuted datasets from (4) that is larger than the observed *HS* statistic from (3).
6. Repeat the analysis for multiple gene sets and estimate false discovery rates (FDRs) from *p*-values of individual sets using the q-values [18].

To identify significant heterogeneous pathways, we applied our novel computational algorithm, HeteroPath in which the heterogeneity of pathways for a cell type was evaluated. We applied a fixed gene set enrichment analysis which combined the gene expression and pathway data. We tested for bidirectionally perturbed pathways in the KEGG database. We were specifically interested in these bidirectionally perturbed pathways because they are individual pathways which contain a significant number of elements that are upregulated and a significant number of elements which are downregulated. The algorithm iterates through all KEGG pathways and assigns a heterogeneity score to each pathway. The heterogeneity score was generated by summing the absolute t-statistics of genes with sufficient magnitude to determine significant differences in gene expression across multiple tissue types. The t-statistic was used as a distance metric to quantify tissue-specific association between gene expression profiles on a per-gene basis. Therefore, the heterogeneity scores factor in both direction and magnitude of perturbation. Although a t-statistic based heterogeneity score of 0.975 or greater is equivalent to a *p*-value < 0.05, it is not appropriate to calculate a p-value from this statistic because of the small number of genes associated with some pathways. Therefore, a permutation based p-value was estimated in our algorithm. An adjusted p-value is then calculated to control for the false discovery rate. The stringent Benjamini-Hochberg correction method [18] is applied to the raw *p*-values produced from the permutation-based calculation.

### Gene set enrichment analysis methods

To reveal the biological relevance of the gene expression profiles obtained, a comparison study of the microarray data was performed using GSEA [8] and parametric analysis of gene set enrichment (PGSEA) [9]. The GSEA algorithm tests if the distribution of the ranks of genes in the gene set differs from a normal distribution using a weighted Kolmogorov-Smirnov test. PGSEA is an algorithm used to analyze a gene expression data set for enrichment in gene sets, often by testing whether the average fold-change of a gene set is different from zero. Gene enrichment scores for each of the KEGG pathways within each tissue sample were calculated using both GSEA and PGSEA. The GSEA procedure allows for selection of a main parameter. The final output of enriched pathways is affected by the ranking metric which measures the level of difference in gene expression between phenotypes. Therefore, we compared the GSEA results using different ranking parameters and observed a strong overlap among the enriched pathways when a t-test statistic (t-test) or the Pearson correlation coefficient was used for quantitative studies. Since HeteroPath is a t-statistic based algorithm, using the t-statistic quantitative measure for GSEA was more appropriate.

To visualize the degree of heterogeneity identified by different gene set enrichment analysis methods, GSEA, PGSEA, and HeteroPath, we calculated a pathway z-score for significantly differentially expressed pathways identified by the three independent algorithms and generated heatmaps. The Z-score for each pathway was calculated using the PGSEA method [9]. In both microarray data sets, we first calculated the fold change values for every gene by comparing each of the three tissues individually with the median expression across all tissues. Using those fold-change values we next calculated the mean of the total fold change values (μ) and the standard deviation of the total fold change values (σ). We denoted the mean of fold change values for a given pathway as *x*_*p*_ and the number of genes in a given pathway as p, and then calculated the pathway Z score as

$$ Z=\left({x}_p-\mu \right)\ast \frac{p^{1/2}}{\sigma } $$

By calculating the pathway Z score for all significant pathways, we were able to generate individual heatmaps for each of the algorithms and visualize the degree of heterogeneity identified by the significant pathways from each respective algorithm.

### HeteroPath performance evaluation

The performance of HeteroPath was evaluated by calculating receiver operating characteristic (ROC) curves and area under the curve (AUC) values for each dataset at varying fold-change thresholds (1.5, 2, and 3) using the R package pROC [19]. We first defined a list of bona fide true positive and true negative pathways in each dataset by using the gene expression values to identify pathways which either had a q-value < 0.01 (“positive”) or q-value > 0.2 (“negative”) in GSEA and PGSEA. We then drew a ROC curve for distinct fold change threshold values in the HeteroPath algorithm. Since HeteroPath identifies significantly differentially expressed genes in each tissue sample by comparing each tissue with the median gene expression of all tissues, varying the fold change threshold influences the number of genes included to calculate the heterogeneity score for each pathway. Using the heterogeneity score, we identified the HeteroPath enriched pathways and compared them to the true positives and true negatives. By calculating the AUCs of the ROC curves based on binary classification of the pathways to the ground truth at three distinct fold change thresholds we were able to evaluate the performance of HeteroPath. We further evaluated the significance of the AUC values by performing a permutation test. By randomly permuting the class labels and running HeteroPath, we recorded AUC values. We repeated the process 1000 times and recorded all the “random” AUCs. Finally, we compared the observed AUC with the empirical distribution of “random” AUCs from the permutation tests to obtain a *p*-value which is defined by the fraction of “random” AUCs greater than or equal to the observed AUC value.

### Simulations

To further test the validity and performance of HeteroPath we designed a simulation study. The simulation studies were designed using a linear additive model to generate normalized microarray data on m genes and n samples [20]. The samples were divided in three groups representing a scenario involving gene set enrichment analysis for three tissues:

$$ {y}_{ij}={\alpha}_i+{\beta}_j+{\in}_{ij} $$

where *α*_*i*_ ~ N(μ = 0, σ = 1) is a gene-specific effect, such as a probe-effect, with *i* = 1,…, m, *β*_*j*_ ∼ N(μ_j_, σ_j_) is a sample-effect with j = 1, 2, 3 and ∈_*ij*_ ∼ N(μ = 0, σ = 1) corresponds to random noise.

To assess statistical power and false positive rate (type-I error), we designed a microarray gene expression data set with m = 5000. Next, we simulated two differently sized differentially expressed gene sets. The first containing 50 genes and the second containing 150 genes. We considered different numbers of samples, *n* = 10, 20, 40, 60, and varying conditions leading to different simulation scenarios for each gene set size. We performed the simulation study varying fractions of differentially expressed genes in the gene set (25, 50 and 80%) and varying the signal-to-noise ratio (the magnitude of the mean sample effect in differentially expressed genes for one of the sample groups).

In the differentially expressed genes scenario, for *β*_*j*_, we set μ_1_ = 0.5, μ_2_ = μ_3_ = 0 for the weak effect; μ_1_ = 1, μ_2_ = μ_3_ = 0 for the strong effect; and σ_1_ = 0.5, σ_2_ = σ_3_ = 1 for both cases. For the non-differentially expressed genes case we set μ_1_ = μ_2_ = μ_3_ = 0 with σ_1_ = σ_2_ = σ_3_ = 1.

We simulated 500 independent data sets using these parameters. For each of the gene set enrichment methods we generated an enrichment score matrix for both gene sets (differentially expressed and non-differentially expressed). We then performed an ANOVA on the score matrix for the two gene sets for a difference in mean between the three groups of samples at a significance level α = 0.05. Across the 500 simulations, we estimated the statistical power as 1 minus the ratio of non-rejections of the differentially expressed gene set and the empirical type-I error as the ratio of rejections of the non-differentially expressed gene set at a significance level α = 0.05.

### Annotated transcriptional regulators of heterogeneous genes

To identify sets of unique transcription factors associated with heterogeneous pathways, we searched for transcription factors that have been experimentally proven by ChIP-seq to bind annotated motifs from the ENCODE project [21] in the promoter regions of the heterogeneous genes. Our goal was to assess whether the motif is statistically over-represented in the set of DNA sequences of the heterogeneous genes from a single pathway. The method requires three steps. First, we extracted 2.5 kb upstream of the transcription start site for each of the heterogeneous genes and examined enrichment for transcription factor binding sites (TFBSs) based on the TRANSFAC [22] and JASPAR [23] databases. We then derived a significance score which is a comparison of the enriched motif found in the set of upstream sequences as compared to a randomly selected set of sequences. In order to calculate significance, we first needed to identify the probability distributions of TFBS for a single transcription factor between the heterogeneous gene set and the randomly selected genes in the mouse genome. Then, we derived a *p*-value for the number of TFBS using the randomly selected background sequence set, which explains the probability of obtaining the number of TFBS observed merely by chance. Low *p*-values (*p* < 0.05) suggested that the motif was significantly over-represented and likely has a biologically relevant function. By identifying enriched motifs within each of the heterogeneous pathways, we further uncovered the transcription factors associated with tissue-specific endothelial cell signaling pathways. We also confirmed that these transcription factors were expressed in the respective tissue.

### Putative regulatory transcription factors

We further predicted which transcription factors regulate the heterogeneous gene expression between distinct cell types by identifying transcription factors predicted to bind the overrepresented motifs. This was performed by scanning the promoter regions of the heterogeneous genes and assessing the propensity of a transcription factor to bind a given sequence based on the PWM scores obtained from TRANSFAC [22] and JASPAR [23, 24]. We performed this analysis using the MATCH algorithm [25]. This search algorithm uses a matrix similarity score (MSS) and a core similarity score (CSS) to measure the quality of the match between the PWM score and the sequence. This score ranges between 0.0–1.0. If the score is above 0.7, we consider these as putative regulatory transcription factors.

The MATCH algorithm has tunable cutoffs that allow for minimizing the false negative rate (minFN), minimizing the false positive rate (minFP), and minimizing the sum of both errors (minSum). We utilized the minSum cutoff which computes a sum of both false positive and false negative rates to find cut-offs that give an optimal number of false positives and false negatives. The number of matches found in the exon sequences for each matrix is computed using minFN cutoffs which define 100% of false positives. The sum of percentages for false positives and false negatives is then computed for every cut-off ranging from minFN to minFP. The minimum sum cut-off is then defined as the minSum cut-off.

### Constructing gene regulatory networks

The known and putative gene regulatory networks were reconstructed in R using the RTN package [26] for visualization. This computational framework establishes interactions and structure of the network by mapping the interactions between upregulated transcription factors identified through motif enrichment and their respective heterogeneous genes identified by HeteroPath.

### Pairwise differential gene expression

In a separate analysis, prior to applying our novel method of identifying heterogeneous pathways, we first performed hierarchical clustering and generated tree plots to evaluate the differential expression across the studied datasets (Additional file 1: Figure S1). Further, to assess the level of heterogeneity among the tissues being analyzed we identified individual differentially expressed genes using *limma*. We compared the gene expression among three of the endothelial cell tissues and among three of the neuronal cell tissues to identify the heterogeneity of endothelial cells and the heterogeneity of neuronal cells. To address the degree of differential expression, we assigned confidence intervals to the differential expression. The transcripts were ranked based on the degree of differential expression using the fold-change (FC) in expression level metric [27, 28]. These statistics were computed using the biological replicates and the variance between the replicates to assign a probability value that indicates an incorrect classification of a gene as being differentially regulated. These statistical techniques allowed for a robust analysis that iterates through the transcriptomic cohort to identify genes that are differentially expressed. Gene expression differences were assessed in *limma* with false discovery rate (FDR) correction for multiple testing [29]. Genes with an adjusted *p* ≤ 0.05 and a FC ≥ 2 were considered significantly differentially expressed. This analysis did not allow us to sufficiently understand the underlying heterogeneity biology therefore we sought out to elucidate characteristic pathways explaining the heterogeneity.

## Results

### Identification of heterogeneously expressed tissue-specific pathways

First, we used a parametric and a non-parametric gene set enrichment analysis, PGSEA [9] and GSEA [8] respectively, as gene set enrichment methodologies followed by our novel algorithm HeteroPath to analyze organ-specific endothelial and tissue-specific neuronal transcriptomics data (Fig. 1a). In both datasets we evaluated three distinct tissues with a well-balanced coverage of three samples per tissue. PGSEA identified differentially expressed gene sets by testing whether the average expression of genes in a gene set deviates from the overall expression of all genes in the sample. GSEA aims to test the up- or downregulation of gene sets by testing the expression levels of individual genes. In this type of analysis, no threshold is set to select for significantly differentially expressed genes, but rather all genes are used to determine the differential expression of the pathway. Furthermore, GSEA makes the assumption that the more differentially expressed a gene is, the more biological relevance it has. We implemented our novel algorithm HeteroPath which assigns a heterogeneity score to each pathway based on how distinct its elements are across all tissues versus a “virtual median cell type”, i.e. the virtual endothelial cell that represents the median of endothelial cells from all tissues. It attributes a higher heterogeneity score to the pathways containing the most heterogeneously expressed genes when comparing distinct tissues independent of cumulative upregulation or downregulation (Fig. 1b). For example, if three elements of a pathway are similarly upregulated and three elements of pathway are also similarly downregulated in a given tissue when compared to the gene expression of the virtual median cell, HeteroPath would rank this as a highly heterogeneous pathway while PGSEA and GSEA would not consider this as a significant pathway.

### Metrics of the HeteroPath algorithm

Two comprehensive studies were conducted to assess the performance of our novel pathway-based algorithm to detect tissue-specific gene regulatory networks. In the first study, we analyzed endothelial cells from three out of nine mouse organs. Each sample consisted of 28,815 probes that were mapped to the 186 KEGG pathways. All of the KEGG pathways were evaluated in the analysis to simulate the differential expression change for all annotated biological processes. More specifically, the fold change in differential expression was drawn from a normal distribution with the mean set at 1.5, 2, 3 and the standard deviation at 0.5. In the second study with neuronal cell populations, all of the parameters were the same, with the only difference being that there were 22,690 probes representing the genome. The performance of the HeteroPath algorithm was evaluated by calculating the receiver operating characteristic (ROC) and area under the curve (AUC) values for each dataset using the R package pROC.

**Fig. 2:**
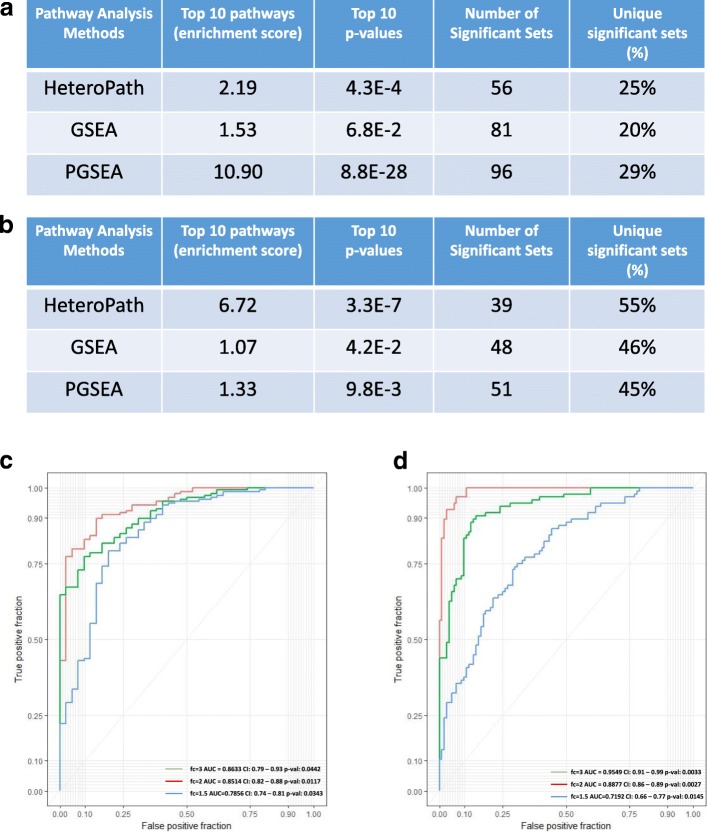
Comparison of enriched pathways **a** The significantly enriched experimental sets and canonical pathways in mouse endothelial cells were inferred by HeteroPath, GSEA, and PGSEA. Top 10 enrichment scores, p-values, numbers of significant gene sets, and percentage of unique gene sets are shown. **b** The significantly enriched experimental sets and canonical pathways in mouse neurons were inferred by HeteroPath, GSEA, and PGSEA. Top 10 enrichment scores, p-values, numbers of significant gene sets, and percentage of unique gene sets are shown. **c** ROC curves for the HeteroPath algorithm using the endothelial cell dataset. fc = fold-change; AUC= area under curve.  **d** ROC curves for the HeteroPath algorithm using the neurons dataset fc = fold-change; AUC= area under curve

In the endothelial cell heterogeneity study, the results showed that all three algorithms identified significantly enriched gene sets to distinguish the three endothelial cell populations. Furthermore, HeteroPath identified the least number of significant sets while PGSEA identified the largest number of significant sets (Fig. 2a). Of the significant sets identified, only 20% of the significant sets identified were unique to the GSEA algorithm while 25 and 29% were unique to HeteroPath and PGSEA, respectively. PGSEA demonstrated a less stringent functional class scoring technique with significantly higher enrichment scores and more significant *p*-values (Fig. 2a). In the study of neuronal heterogeneity (Fig. 2b), HeteroPath obtained the highest enrichment score, most significant p-values, and highest percentage of unique significant sets (55%). These results suggest that HeteroPath performs more optimally when the heterogeneity of pathways is not unidirectional but includes upregulated and downregulated genes when compared to the virtual median cell type and may thus reflect a tight regulation of pathways.

The AUC values ranging from 0.7856 to 0.8633 in the endothelial study (Fig. 2c) and 0.7192 to 0.9549 in the neuronal study (Fig. 2d) indicate that the power of the HeteroPath algorithm increased as the total proportion of genes increased and the fold change increased. Importantly, the HeteroPath algorithm increased in power significantly in the neuronal dataset because it contained a higher number of differentially expressed genes.

### Comparison of methods using simulated data

HeteroPath is a pathway-based algorithm which yields tissue-specific enrichment scores. Therefore, we evaluated the statistical power and type I error of HeteroPath, PGSEA and GSEA using simulated data. We simulated microarray data using a linear additive model with sample and probe effects for 5000 genes and three groups of samples (see Methods for details). Using simulated data for each scenario, we calculated the pathway enrichment scores using HeteroPath, PGSEA, and GSEA. For the differentially expressed gene set, we estimated the statistical power for each method as a function of the sample size. At the same time, for the non-differentially expressed gene set, we estimated the empirical type-I error rate. The results of this simulation (Additional file 1: Figs. S2 & S3) illustrate that HeteroPath performs with comparable statistical power while maintaining similar control of the type-I error rate when compared to GSEA and PGSEA.

### Endothelial heterogeneity

**Fig. 3:**
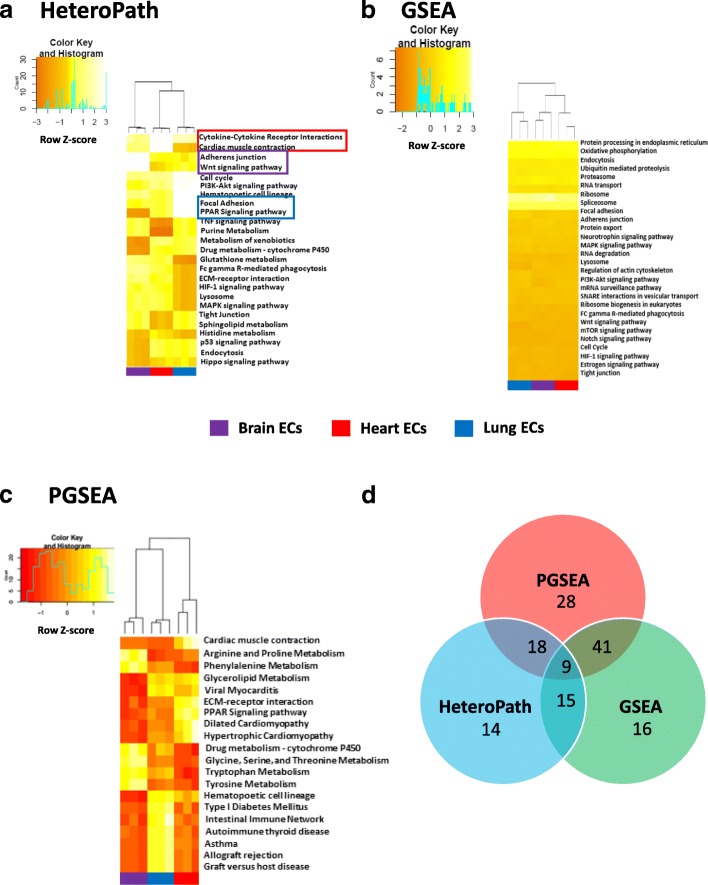
Endothelial cell heterogeneity. **a** Heat map of heterogeneous pathways identified by HeteroPath from Brain, Lung, and Heart endothelial cells. The orange to yellow to white gradient represents increasing expression of the pathway with orange representing minimal expression while the white represents high expression of the pathway. Upregulated tissue-specific pathways are highlighted in colored boxes. **b, c** The results of enriched PGSEA and GSEA pathways from Brain, Lung, and Heart endothelial cells. The orange to yellow to white gradient represents increasing expression of the pathway with orange representing minimal expression while the white represents high expression of the pathway. **d** A Venn diagram displaying the number of overlapping and unique KEGG pathways identified by HeteroPath, PGSEA, and GSEA

In order to assess the degree of organ-specific endothelial heterogeneity we applied the three distinct functional class scoring algorithms to freshly isolated mouse endothelial cells from several organs. The results obtained from the HeteroPath algorithm display the most heterogeneous pathways in endothelial cells for three of the nine vascular beds studied. The heterogeneous pathways were ranked in the order of largest to smallest heterogeneity score where only top statistically significant pathways are shown (Fig. 3a). The most prominent upregulated pathways identified using HeteroPath were the “Wnt signaling” and “adherens junction” pathways in brain endothelial cells; “focal adhesion”, “PPAR signaling”, “PI3K-Akt signaling” pathways in lung endothelial cells; and “cardiac muscle contraction” and “cytokine-cytokine receptor interactions” pathways in heart endothelial cells (Fig. 3a). The HeteroPath algorithm further assigns tissue specificity to the heterogeneous pathways when 60% of the heterogeneous elements of the pathway have unique expression in a specific organ (Fig. 3a). GSEA analysis primarily identified molecular pathways involved in regulating global molecular function such as RNA and protein synthesis, processing, and degradation (Fig. 3b) which were statistically significant but often represented minimal or moderate gene expression changes when compared to the virtual median endothelial cell. PGSEA analysis (Fig. 3c) also identified signature pathways that were greater in magnitude than GSEA, but the pathways were distinct from those identified by the HeteroPath algorithm. For example, PGSEA revealed amino acid metabolism pathways as being differentially expressed in brain endothelial cells. This finding likely reflects the importance of individual metabolic enzymes required in brain endothelial cells [30].

Of the 186 KEGG gene sets assessed, PGSEA detected 96 gene sets as statistically significant at *p* < 0.05 in this data set whereas GSEA detected 81 gene set at this significance (Fig. 3d). The *p*-values obtained by PGSEA were generally smaller than p-values of corresponding gene sets obtained by GSEA. These methods specifically target pathways cumulatively regulated in a single direction, but do not consider that tissue-specific heterogeneity which may involve both heterogeneous upregulation and downregulation of elements within a single pathway unlike HeteroPath which ranks overall heterogeneity of a pathway by assessing the cumulative gene expression distance from that of the “virtual median endothelial cell” for each gene within a pathway.

We performed comparative analysis to determine the number of significant sets which were exclusive to a particular algorithm (Fig. 3d). For example, HeteroPath uncovered 14 unique pathways. Furthermore, with consistent thresholds applied to the different functional class scoring techniques, HeteroPath identified the least number of significant sets (56), while GSEA and PGSEA identified 81 and 96 significant sets, respectively (Fig. 3d).

### Neuronal heterogeneity

**Fig. 4:**
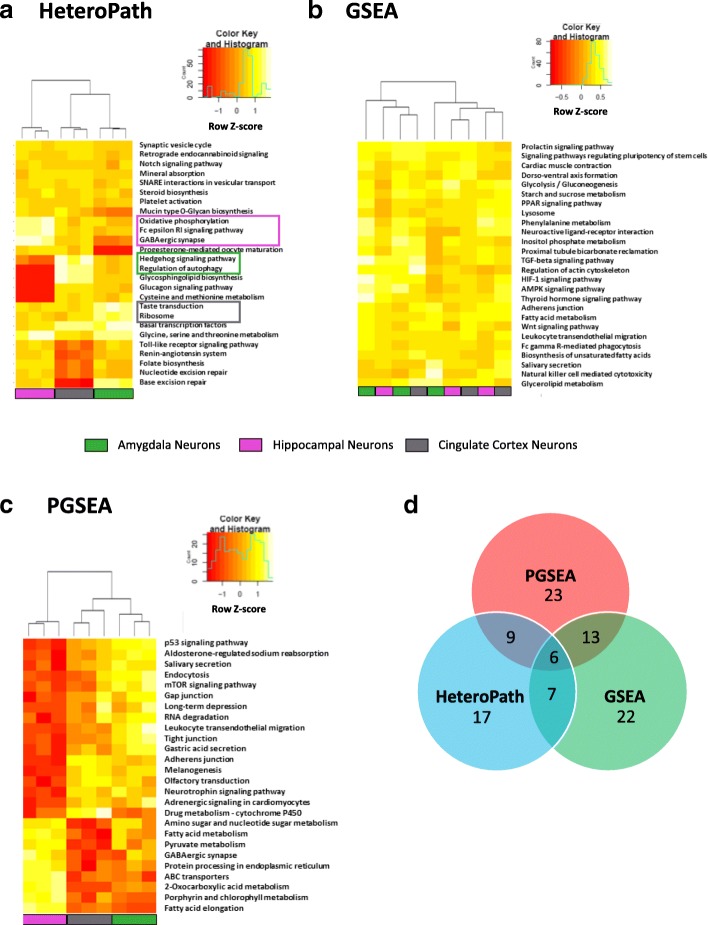
Neuronal heterogeneity **a** Heat map representation of heterogeneous pathways identified by HeteroPath from hippocampal, cingulate cortex, and amygdala neurons. The orange to yellow to white gradient represents increasing expression of the pathway with orange representing minimal expression while the white represents high expression of the pathway. Upregulated tissue-specific pathways are highlighted in colored boxes. **b, c** The results of enriched PGSEA and GSEA pathways from hippocampal, cingulate cortex, and amygdala neurons. The orange to yellow to white gradient represents increasing expression of the pathway with orange representing minimal expression while the white represents high expression of the pathway. **d** A Venn diagram displaying the number of overlapping and unique KEGG pathways identified by HeteroPath, PGSEA, and GSEA

To assess the relative fidelity of neuronal heterogeneity we applied HeteroPath, GSEA, and PGSEA to neurons isolated from 12 regions of the mouse forebrain [31]. To perform a comparison between the three algorithms, we focused on three distinct regions namely the hippocampus, the cingulate cortex, and the amygdala. The results showed large statistical differences between the three independent algorithms which emphasizes the fundamental molecular difference between neurons at their basal state in various regions of the brain. The HeteroPath algorithm identified the most distinct tissue-specific pathways among the three neuronal populations. For instance, hippocampal neurons exhibited an upregulation of “oxidative phosphorylation” and “GABAergic synapse”; cingulate cortex neurons basally upregulated “Hedgehog signaling” and “regulation of autophagy”; while, amygdala neurons upregulated “taste transduction” and “ribosome” (Fig. 4a). In the case of GSEA analysis, the subsets of neurons in distinct regions of the brain exhibited similar molecular signatures (Fig. 4b). Across entire gene sets, there were no tissue-specific pathways. In fact, the algorithm was unable to cluster the neuronal populations into distinct groups. PGSEA, on the other hand, was able to differentiate the three different populations of neurons and identify pathways which contained several upregulated genes in a single tissue (Fig. 4c). PGSEA primarily identified pathways in which there was a significant upregulation in one of the tissues relative to the downregulation in the other tissues. For instance, the entire fatty acid metabolism pathway was downregulated in the cingulate cortex and amygdala neuron populations and hence upregulated in the hippocampal neurons (Fig. 4c).

In the analysis of neuronal cellular heterogeneity, using the three independent algorithms showed that identifying tissue-specific pathways requires prioritizing the up- and downregulation of individual genes within a single pathway. PGSEA and GSEA detected similar numbers of significantly differentially regulated gene sets while HeteroPath detected the least number of differentially expressed pathways (Fig. 4d), but these pathways were able to segregate the neuronal populations most distinctively and thus elucidate pathways descriptive of each neuronal subpopulation. In addition, more than half of the significant sets identified by GSEA and PGSEA overlapped while HeteroPath detected 17 unique pathways which likely contributed to the distinctive clustering of the neuronal subpopulations (Fig, 4d).

### Tissue-specific gene regulatory networks

Based on the design of the HeteroPath algorithm, each heterogeneous pathway reflected the simultaneous upregulation and downregulation of several member genes within a pathway in each tissue. To visualize the role of each significant heterogeneous element within one of the brain endothelium-specific pathways and one of the hippocampal neuronal pathways, we generated respective gene expression heat maps for Wnt signaling and oxidative phosphorylation.

**Fig. 5:**
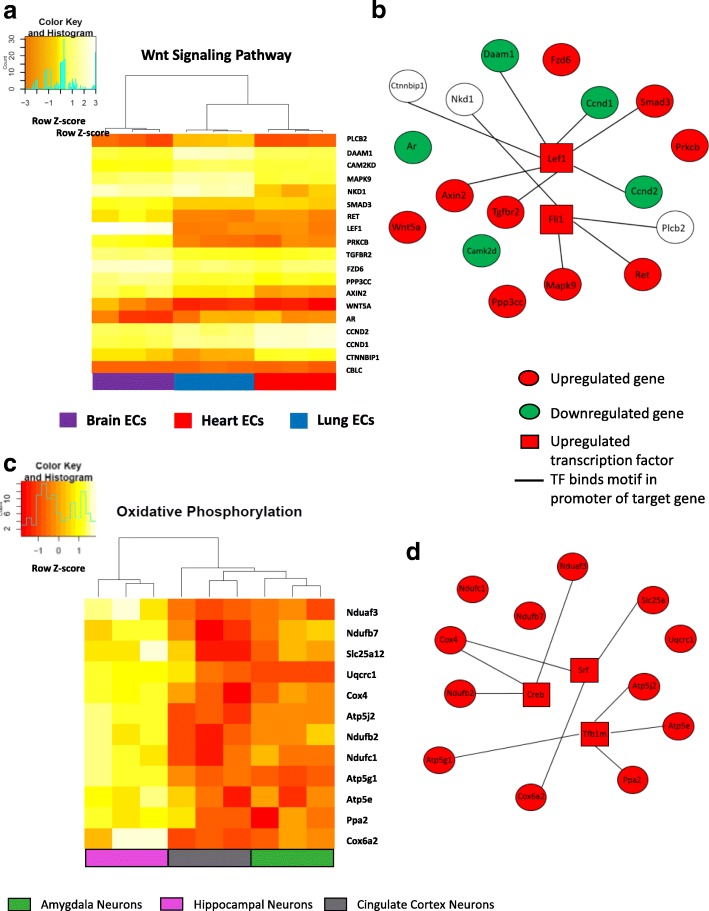
Gene regulatory networks for HeteroPath tissue-specific pathways **a** The heat map shows the normalized mRNA expression level in Brain, Lung, and Heart endothelial cells for the heterogeneous genes of the Wnt signaling pathway. **b** Wnt signaling gene regulatory network including upregulated transcription factors which bind motifs in the promoter region of brain-specific heterogeneous elements. **c** The heat map shows the normalized mRNA expression level in hippocampal, cingulate cortex, and amygdala neurons for the heterogeneous genes of the oxidative phosphorylation pathway **d** Oxidative phosphorylation gene regulatory network including upregulated transcription factors which bind motifs in the promoter region of hippocampal-specific heterogeneous elements

Using the heterogeneous elements in the Wnt signaling pathway (Fig. 5a), we examined the role of putative transcription factors responsible for the brain endothelial cell specific gene expression signature by identifying transcription factors which have been experimentally proven as identified by the ENCODE database [21] to bind motifs in the promoter regions of the heterogeneously expressed genes (Additional file 1: Figure S4A). For the Wnt signaling pathway, lymphoid enhancer-binding factor 1 (LEF1) and friend leukemia integration 1 (FLI1) were the top candidate transcription factors (Additional file 1: Figure S4A). The Wnt signaling gene regulatory network (Fig. 5b) contains upregulated genes in brain endothelial cells such as LEF1, Wnt family member 5A (WNT5A), transforming growth factor beta receptor 2 (TGFBR2), and Axin-related protein (AXIN2) as well as downregulated genes such as cyclin D1 (CCND1) and cyclin D2 (CCND2) (Fig. 4a).

By examining the tissue-specific neuronal pathways, we identified oxidative phosphorylation as a key upregulated pathway in the hippocampal neurons. Analysis of the heterogeneous elements in the oxidative phosphorylation pathway demonstrated that the high heterogeneity score was driven by the significant upregulation of cytochrome c oxidase family genes as well as mitochondrial ATP synthase genes in hippocampal neurons (Fig. 5c). Using analogous methods to uncover regulatory transcription factors, we identified three central transcription factors which may drive the upregulation of oxidative phosphorylation in hippocampal neurons: cAMP response element binding protein (CREB), serum response factor (SRF), and Dimethyladenosine transferase 1, mitochondrial (TFB1M) (Additional file 1: Figure S4B). From these results, we generated a hippocampal neuron specific gene regulatory network which included the regulatory transcription factors and the oxidative phosphorylation heterogeneous genes (Fig. 5d).

## Conclusions

Organ-specific endothelial cells and tissue-specific neuronal cells display remarkable cellular heterogeneity in both their genotypic and phenotypic characteristics [11]. Although it is well established that phenotypes of cell populations in different regions are distinct, the unique transcriptomic signatures that define cellular heterogeneity are less clear. Evaluating tissue-specific gene expression is critical for identifying tissue-specific mechanisms of disease [32]. Here we used the transcriptomic analysis of mouse endothelial cells from distinct tissues [4] and neuronal cells from regions of the mouse forebrain to identify signature gene regulatory networks.

Since endothelial cells are extraordinarily plastic and are known to change their phenotype in culture [33], the data from these freshly isolated endothelial cell populations was particularly relevant for identifying tissue-specific endothelial pathways [4]. It is also known that regionalization in the brain is directed by molecular gradients during development [34]. The degree to which this regionalization causes neurons to express genes heterogeneously was previously unknown. In our work, we observed that the gene expression distance between hippocampal, cingulate cortex, and amygdala neurons isolated from mouse forebrain significantly surpassed the range of the expression distances between replicate experiments, thus indicating gene expression profile specificity for each of the studied brain regions. One of the major challenges in neurobiology has been to create signature gene regulatory networks which are associated with cell type specific phenotypes such as morphology, firing patterns, connectivity and synaptic transmission [31].

To address this challenge in both endothelial cells and neurons, we developed the computational algorithm HeteroPath which performs a contextual analysis by assigning a higher heterogeneity score if multiple elements are heterogeneous within a single pathway. Furthermore, this computational model suggests experimentally testable predictions for understanding the general architecture of the gene regulatory networks that establish how basal cellular identity is maintained [35].

In this study, our objective was to design an algorithm, which first identified heterogeneously expressed pathways in cell populations of unique organs or tissues. The key principle in our analysis was that we determined a pathway heterogeneity score which allowed for individual elements of the pathway to be either upregulated or downregulated when compared to the median of all tissues.

In order to show the application of identifying heterogeneous pathways, transcription factors and gene regulatory networks were generated for HeteroPath, GSEA, and PGSEA. When comparing the novel HeteroPath analysis with PGSEA [9], which ranks genes according to their relative expression levels without prior identification of heterogeneous pathways, we found that HeteroPath uniquely identified signature gene regulatory networks for defining tissue specificity. Thus, the HeteroPath approach is well-suited for identifying tissue-specific druggable signaling targets or regulatory signaling pathways because it particularly identifies tissue-specific regulated pathways. Furthermore, the HeteroPath analysis differs from GSEA because GSEA ranks pathways by cumulative perturbation of genes in a pathway but does not consider the extent of differential expression for each element within the pathway in establishing or maintaining tissue-specific heterogeneity. GSEA primarily identified minimally differentially expressed pathways as tissue specific in some cases and was unable to identify any tissue-specific pathways in other cases. Therefore, GSEA analysis of tissue specificity may be more appropriate for assessment of global cellular quiescence or activity as a function of subtle gene expression changes in distinct tissues.

Traditional over-representation analysis (ORA) methods such as Fisher’s exact test treat genes in a gene set or a pathway simply as gene labels with equal importance, and then test the significance of the over-representation of the gene set among a list of interesting genes. In this type of analysis, the magnitude and direction of change are not evaluated and used to identify tissue-specific gene sets. To complement this approach, we designed HeteroPath to calculate a pathway score that factors in the magnitude and direction of change to identify characteristic pathways segregating distinct populations of cells.

A fundamental question in cellular heterogeneity is defining the nature of interactions of cells from different organs or tissues with the underlying parenchymal cells. Recent studies have described an angiocrine mechanism by which the signals from surrounding cell types influence functions of tissue cells such as their growth and differentiation characteristics [36]. It is also likely that specialized signals from a heterogeneous population of cells influence interactions underlying cells such as in the case of endothelial cells, vascular smooth muscle cells and pericytes [37]. Including gene upregulation and downregulation in the analysis along with the extent of differential expression to define tissue-specific gene expression generates comprehensive tissue-specific signatures as opposed to those obtained by existing gene set enrichment analyses based only on cumulative unidirectional gene regulation. Downregulation of specific genes and pathways is essential for the development of tissues such as during mesodermal differentiation when downregulation of Flk1 followed by a later induction of Flk1 expression is required for the formation of cardiac progenitors [38]. In addition, downregulated genes can act as “valves” which maintain low levels of baseline gene expression and enable upregulation as a response to stressors or stimuli.

In brain endothelium, we uncovered the Wnt signaling pathway as being significantly heterogeneous when compared to heart or lung endothelium. Analysis of regulatory transcription factors that could maintain the brain EC specific upregulation of the Wnt signaling pathways allowed us to identify the upregulation of Lef1, which is known to interact with β-catenin and regulate brain vascularization as well as differentiation of the BBB in vivo [39]. Additionally, the Wnt-associated beta-catenin/TCF7 transcriptional complex has been shown to regulate vascular remodeling through the regulation of smooth muscle cell proliferation and EC growth [40–42]. Similarly, the Wnt pathway member and transcription factor FLI1 was also upregulated in brain endothelial cells and is thought to be among the earliest transcription factors involved in endothelial cell development [43].

In hippocampal neurons, we showed the oxidative phosphorylation biological process to be upregulated in a tissue-specific manner compared to cingulate cortex and amygdala neurons. We predicted that CREB, SRF, and TFB1M are crucial transcription factors driving the upregulation of the oxidative phosphorylation process in a tissue-specific manner. In neurons, CREB is known to be phosphorylated under conditions of hypoxia and oxidative stress which suggests that the CREB activation is a survival program during harmful stimuli and may play a role as a cellular form of defense [44]. In addition, the molecular mechanisms underlying SRF-dependent axon growth have been reported in mouse hippocampal neurons [45]. Furthermore, the mitochondrial transcription factor TFB1M has been implicated in cellular systems in which its upregulation induces mitochondrial biogenesis [46].

### Limitations

The identified tissue-specific gene regulatory networks include regulated pathways that would otherwise be overlooked by conventional analysis methods. Even though HeteroPath shows promise in generating tissue-specific gene regulatory networks, there are limitations of our approach that need to be considered. To assess the performance of HeteroPath, we randomly permuted the class labels and ran HeteroPath to calculate confidence intervals and *p*-values for each of the AUC values at distinct fold-change cut-offs. However, the sensitivity and specificity of these regulated pathways identified by HeteroPath could not be compared with GSEA and PGSEA using biological datasets since the method for generating the ground truth relied on GSEA and PGSEA. Therefore, we generated simulated microarray datasets and applied HeteroPath, GSEA, and PGSEA to detect differentially expressed gene sets. From this simulation study, we found that the statistical power and type I error rates for HeteroPath were comparable to existing gene set enrichment analysis methods.

Similar to PGSEA and GSEA, the HeteroPath algorithm relies on existing pathway databases. It is known that the curated pathways share multiple genes or describe similar phenomena [47]. For instance, we found occludin to be highly upregulated in the BBB, but it is not officially curated as a member of the adherens junction pathway even though it is known that occludins regulate adherens junction pathways in the brain endothelium [48]. In the commonly used KEGG pathway database, which we also employed as a pathway reference database for our analysis, pathways often significantly overlap on a molecular and functional level. Therefore, one limitation of our analyses is the accuracy and comprehensiveness of the underlying pathway database curation. To resolve this issue, a dimension reduction machine learning technique could be implemented to curate and filter the pathways from existing databases. Furthermore, expanding existing annotations to include condition-, tissue-, and cell-specific functions for genes and pathways would allow for the prediction of system variation due to factors such as stimuli, mutations, or environmental change [49]. The goal of our analysis and algorithm development was to develop tools that will allow researchers to predict signature gene regulatory networks for cell types, determine putative transcription factors that could drive the heterogeneity and generate novel biological hypotheses. It should be noted that the biological significance of each signature network would need to be confirmed using in vivo gene deletion or gene depletion studies.

In conclusion, we describe herein a computational algorithm which ranks pathways by assigning heterogeneity scores. This technique allowed us to uncover additional endothelial cell and neuronal signature gene regulatory networks for each tissue that would not have been identified by traditional analyses such as GSEA or PGSEA. Even though our analysis focused on comparing two cohorts of cellular heterogeneity: three endothelial cell populations and three neuronal populations, the algorithm can be readily expanded to assessing pathway heterogeneity between several tissues and implemented in any cellular heterogeneity context. Thus, the described computational approach identifying distinct regulatory pathways and druggable therapeutic targets in endothelial and neuronal populations may be of value in understanding the complex heterogeneity of other tissues.

## Additional file

Additional file 1: **Figure S1.** A) Hierarchical clustering of Endothelial cells from 7 mouse organs Intra- and inter-tissue heterogeneity. Tree plot generated via hierarchical clustering of 500 most variable genes across all distinct tissue endothelial cell samples B) Hierarchical clustering of Neuronal cells from 5 different regions of the mouse forebrain Intra- and inter-tissue heterogeneity. Tree plot generated via hierarchical clustering of 500 most variable genes across all distinct tissue neuronal cell samples. **Figure S2.** Comparison of statistical power and type-I error rate between HeteroPath, GSEA, and PGSEA for DE Gene Set size of 50 genes. The averaged results of 500 simulations are depicted as function of the sample size on the x-axis, for each of the methods. On the y-axis either the statistical power or the empirical type-I error rate is shown. GSE scores were calculated with each method with respect to two gene sets, one of them differentially expressed (DE) and the other one not. Statistical power and empirical type-I error rates were estimated by performing an ANOVA on the DE and non-DE gene sets, respectively, at a significance level of α = 0.05. **Figure S3.** Comparison of statistical power and type-I error rate between HeteroPath, GSEA, and PGSEA for DE Gene Set size of 150 genes. The averaged results of 500 simulations are depicted as function of the sample size on the x-axis, for each of the methods. On the y-axis either the statistical power or the empirical type-I error rate is shown. GSE scores were calculated with each method with respect to two gene sets, one of them differentially expressed (DE) and the other one not. Statistical power and empirical type-I error rates were estimated by performing an ANOVA on the DE and non-DE gene sets, respectively, at a significance level of α = 0.05. **Figure S4.** A) Enriched Wnt Signaling Motifs from Brain endothelial cells The table shows the five most enriched motifs in ChIP-seq peaks and the associated transcription factors. Significance values and significant *p*-values (*p* ≤ 0.05) are shown. B) Enriched Oxidative Phosphorylation Motifs from Hippocampal Neurons The table shows the five most enriched motifs in ChIP-seq peaks and the associated transcription factors. Significance values and significant p-values (p ≤ 0.05) are shown. (PPTX 1265 kb)

## Abbreviations

AUC: Area under curve
ChIP-Seq: Chromatin Immunoprecipitation Sequencing
GSEA: Gene set enrichment analysis
PGSEA: Parametric analysis of gene set enrichment
ROC: Receiver operating characteristic
TFBS: Transcription factor binding site
