# Supplementary material for: A computational approach to identify cellular heterogeneity and tissue-specific gene regulatory networks
Source: BMC Bioinformatics. 2018 Jun 7;19:217. doi: 10.1186/s12859-018-2190-6 (PMC6019795; doi:10.1186/s12859-018-2190-6)
Supplement: Supplementary file 1 — Figure S1. A) Hierarchical clustering of Endothelial cells from 7 mouse organs Intra- and inter-tissue heterogeneity. Tree plot generated via hierarchical clustering of 500 most variable genes across all distinct tissue endothelial cell samples B) Hierarchical clustering of Neuronal cells from 5 different regions of the mouse forebrain Intra- and inter-tissue heterogeneity. Tree plot generated via hierarchical clustering of 500 most variable genes across all distinct tissue neuronal cell samples. Figure S2. Comparison of statistical power and type-I error rate between HeteroPath, GSEA, and PGSEA for DE Gene Set size of 50 genes. The averaged results of 500 simulations are depicted as function of the sample size on the x-axis, for each of the methods. On the y-axis either the statistical power or the empirical type-I error rate is shown. GSE scores were calculated with each method with respect to two gene sets, one of them differentially expressed (DE) and the other one not. Statistical power and empirical type-I error rates were estimated by performing an ANOVA on the DE and non-DE gene sets, respectively, at a significance level of α = 0.05. Figure S3. Comparison of statistical power and type-I error rate between HeteroPath, GSEA, and PGSEA for DE Gene Set size of 150 genes. The averaged results of 500 simulations are depicted as function of the sample size on the x-axis, for each of the methods. On the y-axis either the statistical power or the empirical type-I error rate is shown. GSE scores were calculated with each method with respect to two gene sets, one of them differentially expressed (DE) and the other one not. Statistical power and empirical type-I error rates were estimated by performing an ANOVA on the DE and non-DE gene sets, respectively, at a significance level of α = 0.05. Figure S4. A) Enriched Wnt Signaling Motifs from Brain endothelial cells The table shows the five most enriched motifs in ChIP-seq peaks and the associated transcription factors. [file 12859_2018_2190_MOESM1_ESM.pptx]

## Slide 1
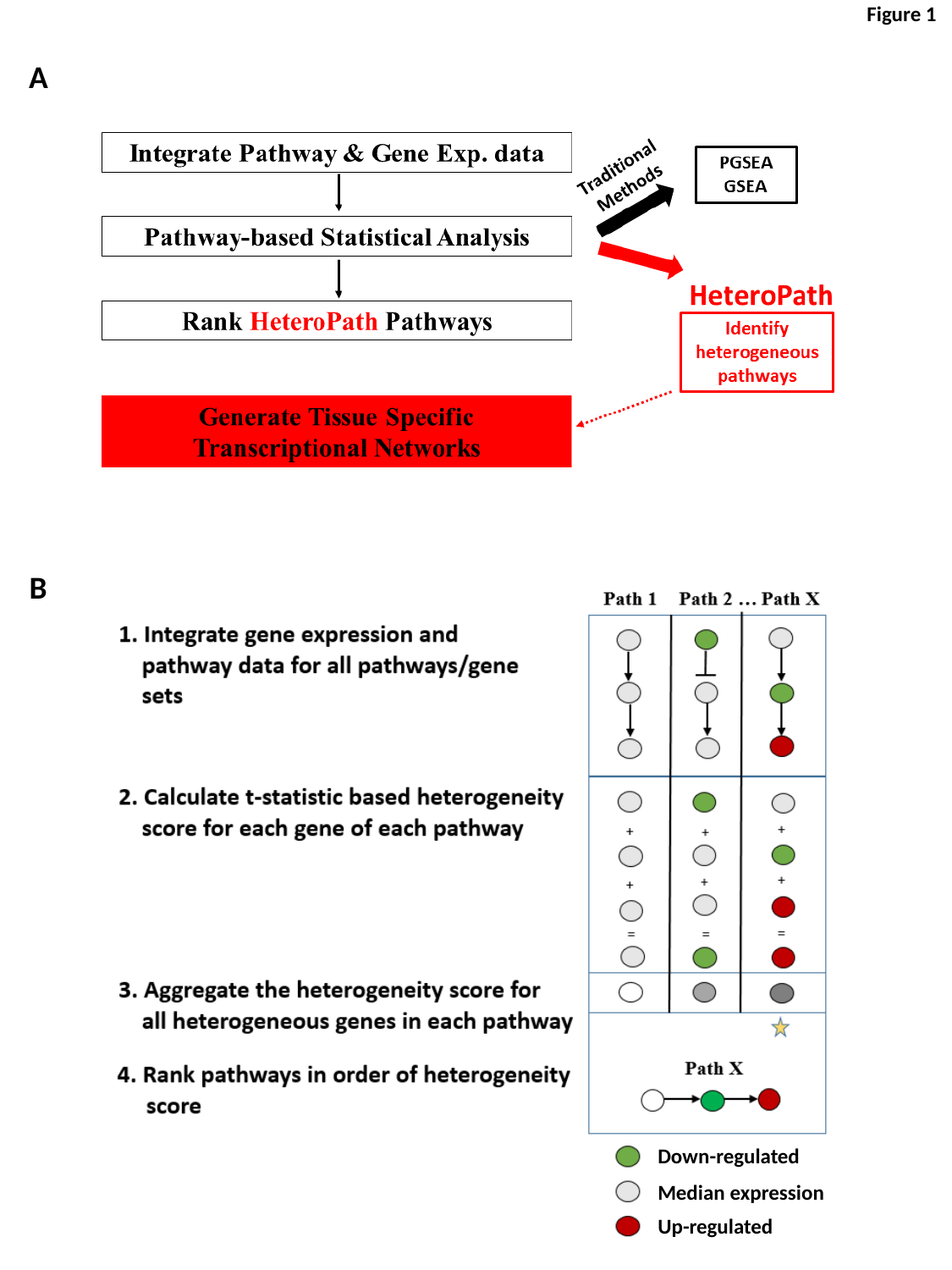

Figure 1
A
B
Down-regulated
Median expression
Up-regulated

## Slide 2
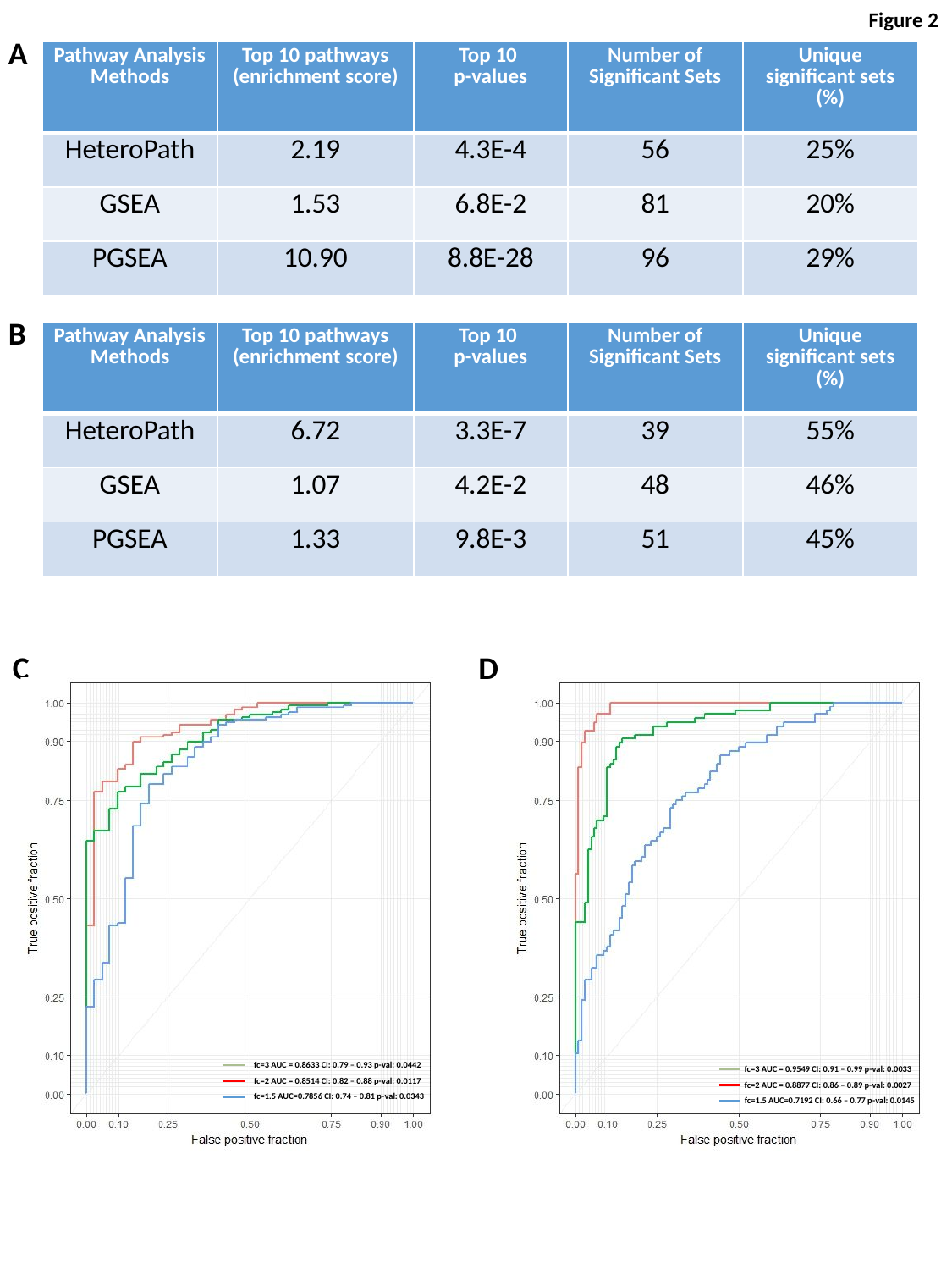

Figure 2
A
| Pathway Analysis Methods | Top 10 pathways (enrichment score) | Top 10 p-values | Number of Significant Sets | Unique significant sets (%) |
| --- | --- | --- | --- | --- |
| HeteroPath | 2.19 | 4.3E-4 | 56 | 25% |
| GSEA | 1.53 | 6.8E-2 | 81 | 20% |
| PGSEA | 10.90 | 8.8E-28 | 96 | 29% |
B
| Pathway Analysis Methods | Top 10 pathways (enrichment score) | Top 10 p-values | Number of Significant Sets | Unique significant sets (%) |
| --- | --- | --- | --- | --- |
| HeteroPath | 6.72 | 3.3E-7 | 39 | 55% |
| GSEA | 1.07 | 4.2E-2 | 48 | 46% |
| PGSEA | 1.33 | 9.8E-3 | 51 | 45% |
C
D
fc=3 AUC = 0.8633 CI: 0.79 – 0.93 p-val: 0.0442
fc=2 AUC = 0.8514 CI: 0.82 – 0.88 p-val: 0.0117
fc=1.5 AUC=0.7856 CI: 0.74 – 0.81 p-val: 0.0343
fc=3 AUC = 0.9549 CI: 0.91 – 0.99 p-val: 0.0033
fc=2 AUC = 0.8877 CI: 0.86 – 0.89 p-val: 0.0027
fc=1.5 AUC=0.7192 CI: 0.66 – 0.77 p-val: 0.0145

## Slide 3
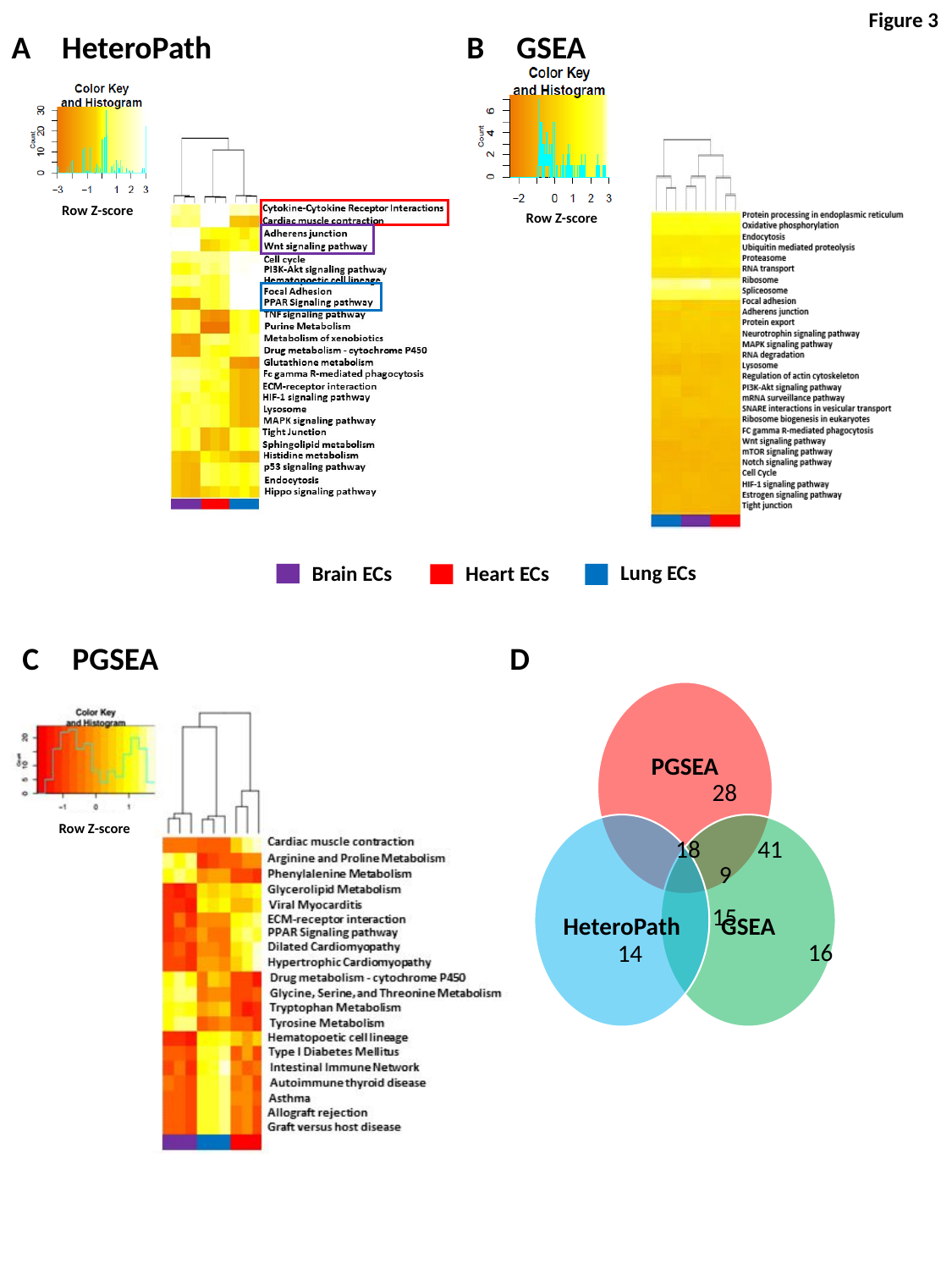

Figure 3
A
HeteroPath
B
GSEA
Row Z-score
Row Z-score
Lung ECs
Brain ECs
Heart ECs
C
PGSEA
D
28
18
41
9
15
16
14
Row Z-score

## Slide 4
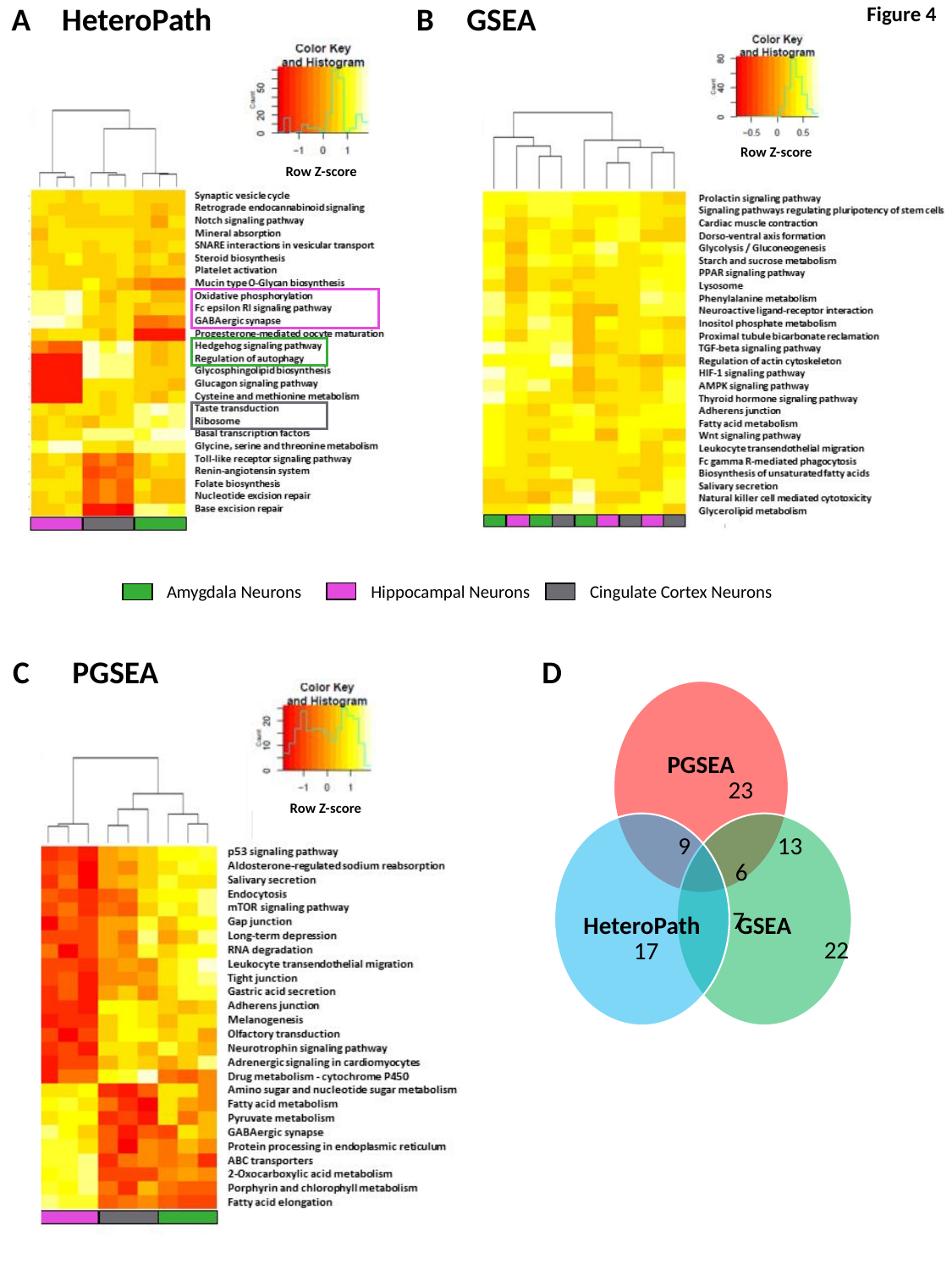

A
HeteroPath
B
GSEA
Figure 4
Row Z-score
Row Z-score
Amygdala Neurons
Hippocampal Neurons
Cingulate Cortex Neurons
C
PGSEA
D
23
9
13
6
7
22
17
Row Z-score

## Slide 5
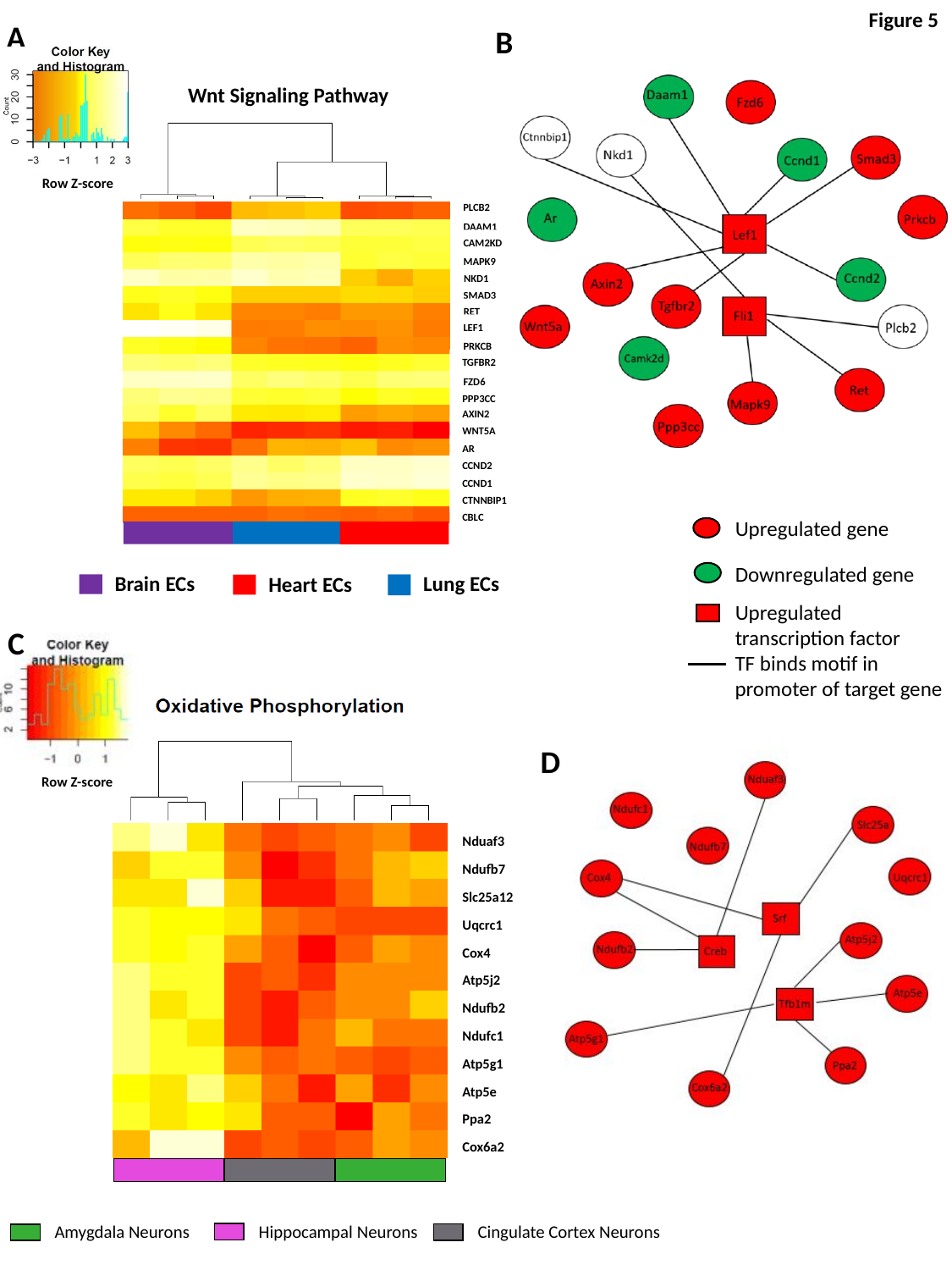

Figure 5
A
B
Row Z-score
Wnt Signaling Pathway
Row Z-score
PLCB2
DAAM1
CAM2KD
MAPK9
NKD1
SMAD3
RET
LEF1
PRKCB
TGFBR2
FZD6
PPP3CC
AXIN2
WNT5A
AR
CCND2
CCND1
CTNNBIP1
CBLC
Upregulated gene
Downregulated gene
Upregulated transcription factor
TF binds motif in promoter of target gene
Lung ECs
Brain ECs
Heart ECs
C
Nduaf3
Ndufb7
Slc25a12
Uqcrc1
Cox4
Atp5j2
Ndufb2
Ndufc1
Atp5g1
Atp5e
Ppa2
Cox6a2
D
Row Z-score
Amygdala Neurons
Hippocampal Neurons
Cingulate Cortex Neurons

## Slide 6
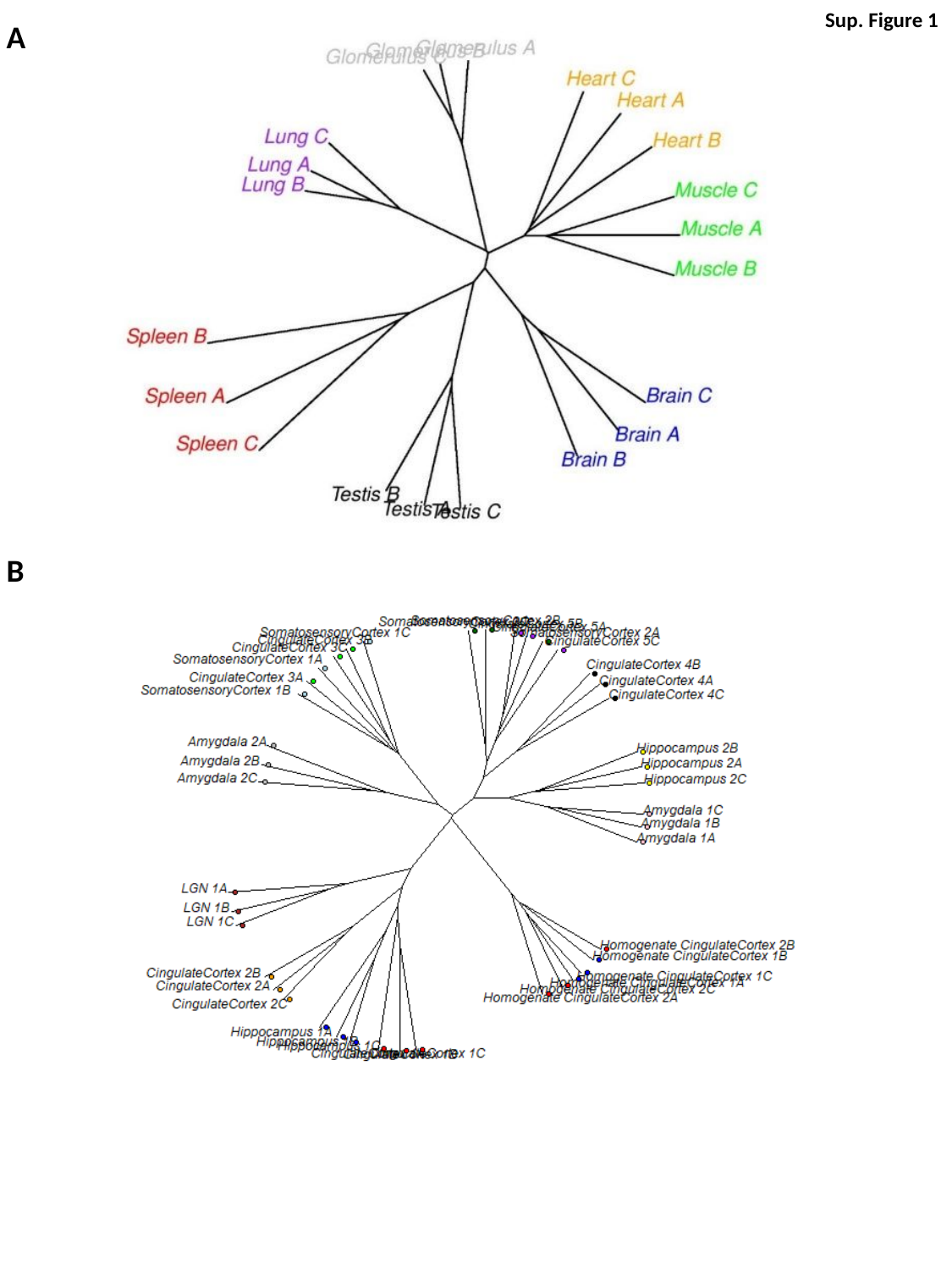

Sup. Figure 1
A
B

## Slide 7
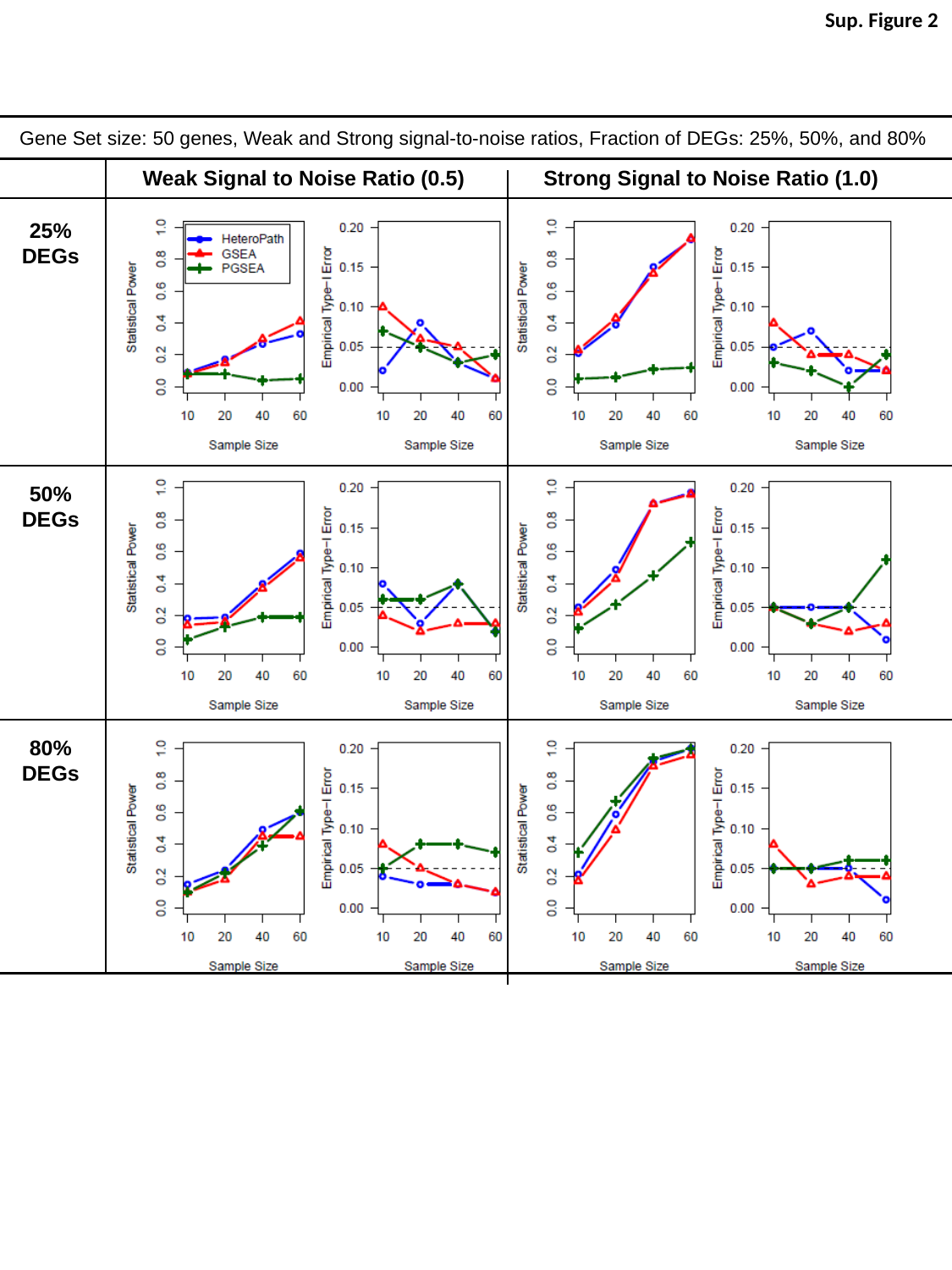

Sup. Figure 2
Gene Set size: 50 genes, Weak and Strong signal-to-noise ratios, Fraction of DEGs: 25%, 50%, and 80%
Weak Signal to Noise Ratio (0.5)
Strong Signal to Noise Ratio (1.0)
25%
DEGs
50%
DEGs
80%
DEGs

## Slide 8
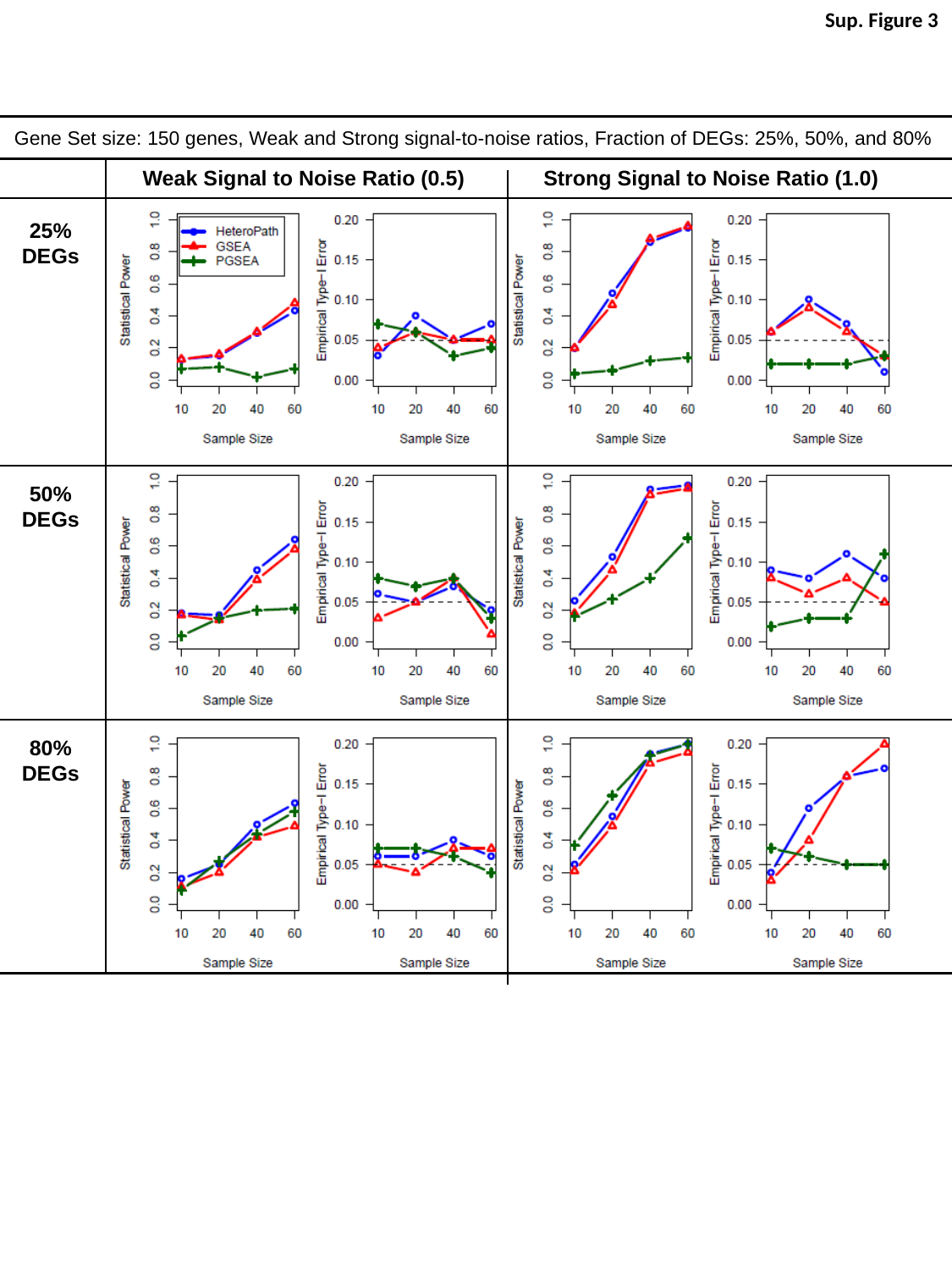

Sup. Figure 3
Gene Set size: 150 genes, Weak and Strong signal-to-noise ratios, Fraction of DEGs: 25%, 50%, and 80%
Weak Signal to Noise Ratio (0.5)
Strong Signal to Noise Ratio (1.0)
25%
DEGs
50%
DEGs
80%
DEGs

## Slide 9
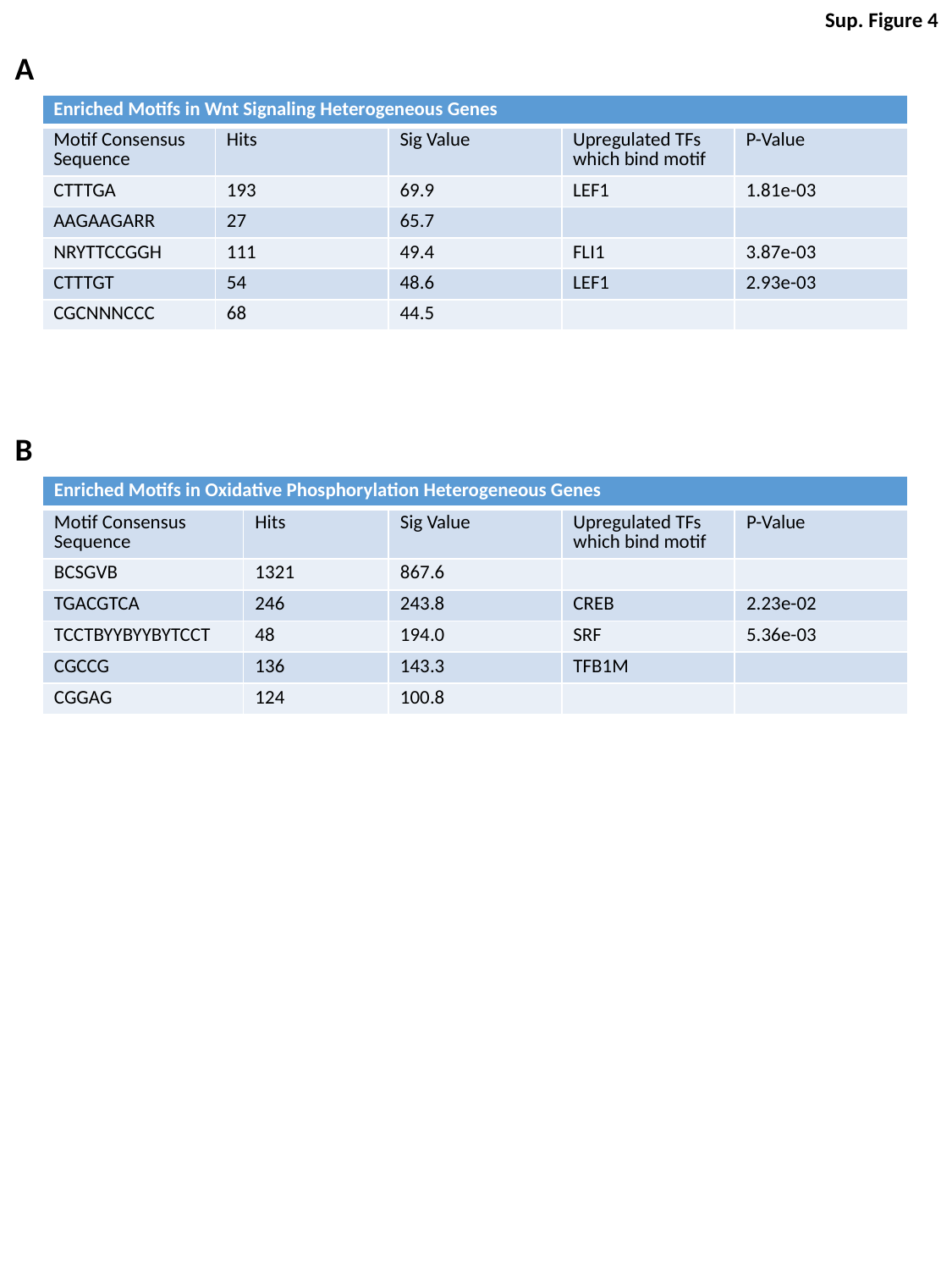

Sup. Figure 4
A
| Enriched Motifs in Wnt Signaling Heterogeneous Genes | | | | |
| --- | --- | --- | --- | --- |
| Motif Consensus Sequence | Hits | Sig Value | Upregulated TFs which bind motif | P-Value |
| CTTTGA | 193 | 69.9 | LEF1 | 1.81e-03 |
| AAGAAGARR | 27 | 65.7 | | |
| NRYTTCCGGH | 111 | 49.4 | FLI1 | 3.87e-03 |
| CTTTGT | 54 | 48.6 | LEF1 | 2.93e-03 |
| CGCNNNCCC | 68 | 44.5 | | |
B
| Enriched Motifs in Oxidative Phosphorylation Heterogeneous Genes | | | | |
| --- | --- | --- | --- | --- |
| Motif Consensus Sequence | Hits | Sig Value | Upregulated TFs which bind motif | P-Value |
| BCSGVB | 1321 | 867.6 | | |
| TGACGTCA | 246 | 243.8 | CREB | 2.23e-02 |
| TCCTBYYBYYBYTCCT | 48 | 194.0 | SRF | 5.36e-03 |
| CGCCG | 136 | 143.3 | TFB1M | |
| CGGAG | 124 | 100.8 | | |
